# Perioperative Challenges in a Patient With Sturge–Weber and Obesity Hypoventilation Syndromes

**DOI:** 10.1155/carm/5537894

**Published:** 2026-03-08

**Authors:** Giuseppe Mincolelli, Antonio Izzi, Matteo Luigi Giuseppe Leoni, Vincenzo Marchello, Maria Grazia Di Carlo, Marco Cascella, Andreaserena Recchia, Ruggero Massimo Corso

**Affiliations:** ^1^ Complex Unit of Anaesthesia and Resuscitation II, Fondazione IRCCS Casa Sollievo Della Sofferenza, San Giovanni Rotondo, Foggia, 71013, Italy, operapadrepio.it; ^2^ Università Degli Studi di Cassino e del Lazio Meridionale. Biblioteca Giorgio Aprea, Cassino, Italy; ^3^ Department of Medicine, Surgery and Dentistry, University of Salerno, Baronissi, 84081, Italy, unisa.it; ^4^ Department of Emergency and Critical Care, Anesthesia and Intensive Care Section, Guglielmo di Saliceto Hospital, Piacenza, Italy

**Keywords:** continuous positive airway pressure, high flow nasal oxygen, obesity hypoventilation syndrome, obstructive sleep apnea syndrome, perioperative management, sleep medicine, Sturge–Weber syndrome

## Abstract

**Background:**

The rare concurrence of Sturge–Weber syndrome (SWS) and obesity hypoventilation syndrome (OHS) presents significant anesthetic challenges, with complexity in airway management and perioperative respiratory care.

**Case Presentation:**

A 55‐year‐old man with SWS and OHS underwent elective dental extraction. He was advised to undergo continuous positive airway pressure (CPAP) therapy (10 cmH_2_O) preoperatively. Premedication included intramuscular clonidine (2 μg/kg). Awake fiberoptic intubation was performed under high‐flow nasal oxygen (HFNO) support. Anesthesia was maintained with propofol and remifentanil via target‐controlled infusion. In the PACU, CPAP intolerance led to desaturation (SpO_2_ 80%), which was rapidly corrected with HFNO (50–70 L/min, FiO_2_ 60%–80%), restoring SpO_2_ > 94% within 3 min. The patient was discharged after 24 h of uneventful monitoring.

**Conclusions:**

Awake fiberoptic intubation and the use of HFNO under strict ventilatory monitoring, as a rescue or bridging strategy, can facilitate safe anesthetic management in high‐risk patients with SWS and OHS who are CPAP‐intolerant. This case highlights the importance of multidisciplinary planning and individualized respiratory support strategies.

## 1. Introduction

Sturge–Weber syndrome (SWS), also known as encephalotrigeminal angiomatosis, is a rare nonhereditary neurocutaneous disorder characterized by vascular malformations affecting the brain (leptomeningeal angioma), facial capillary malformations (port‐wine birthmarks), and ocular abnormalities, typically involving the ophthalmic (V1) and maxillary (V2) trigeminal nerve distributions [1]. Common neurologic sequelae include seizures, stroke‐like episodes, hemiparesis, and cognitive impairment, with seizure control being a critical factor influencing neurologic outcomes [1, 2]. Several environmental and physiologic factors can precipitate seizures in patients who are already neurologically vulnerable. Febrile illnesses affecting the ear, nose, and throat, such as ear, tonsil, or adenoid infections, are reported to occur more frequently in these patients and could impair their neurologic status by lowering their seizure threshold, rendering stroke‐like episodes more likely. In addition, soft and bony tissue hypertrophy that can accompany SWS may be indicative of ear, nose, and throat issues, such as sleep‐disordered breathing such as obstructive sleep apnea syndrome (OSAS), which in turn can influence the neurologic status of these patients [1, 2].

Obesity hypoventilation syndrome is defined by the triad of obesity (BMI ≥ 30 kg/m^2^), daytime hypercapnia (PaCO_2_ ≥ 45 mmHg), and sleep‐disordered breathing. Hypoxemia is frequently associated but is not required for diagnosis [3]. The combination of SWS and OHS presents significant anesthetic challenges, such as difficult airway management due to angiomatous lesions, increased bleeding risk, and heightened perioperative respiratory complications.

This case report describes the comprehensive perioperative management of a patient with concurrent SWS and OHS, highlighting the importance of multidisciplinary planning and specialized respiratory support strategies.

## 2. Case Presentation

**FIGURE 1:**
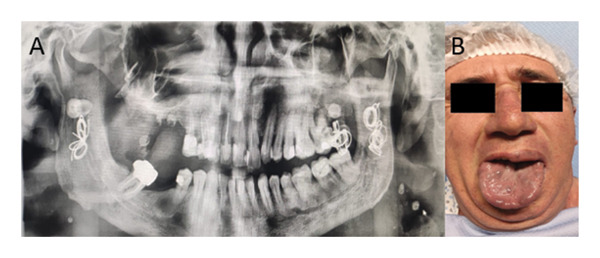
Complex airway involvement in a patient with SWS and OHS with oral and tongue angiomatosis. (A) Panoramic radiograph showing the outcomes of interventional radiologic embolization for oral cavity angiomas following spontaneous hemorrhage, which necessitated an emergency tracheostomy due to a difficult airway. (B) Clinical image depicting multiple vascular angiomas on the posterior surface of the tongue, contributing to significant airway compromise and posing major challenges for intubation.

We present the case of a 55‐year‐old male patient with SWS scheduled for elective dental extraction due to recurrent abscess and infectious complications. His significant medical comorbidities included hypertension, Type 2 diabetes mellitus, obesity with a BMI of 35 kg/m^2^, and severe OSAS with documented poor continuous positive airway pressure (CPAP) compliance. Notably, his surgical history included a previous tracheostomy necessitated by difficult airway management following spontaneous angioma bleeding during a radiological intervention (Figure 1).

### 2.1. Clinical Findings and Preoperative Assessment

Preoperative evaluation revealed characteristic features of OHS, including more severe upper airway obstruction, restrictive pulmonary physiology, and blunted respiratory drive compared to eucapnic obese patients. The patient reported regular follow‐up with sleep medicine specialists and had been diagnosed with OHS two years prior, following persistent hypersomnolence and cardiopulmonary dysfunction. Assessment findings revealed an Epworth Sleepiness Scale (ESS) score of 5, indicating well‐controlled daytime sleepiness without recent exacerbations of hypersomnolence. The patient demonstrated absence of acute pulmonary, cardiovascular, or thromboembolic symptoms and was maintained on current CPAP therapy at 10 cmH_2_O. Preoperative optimization focused on respiratory support continuity and anxiolysis. CPAP continued throughout the perioperative period, including the morning of surgery, with arrangements for machine availability in the postanesthesia care unit (PACU). Premedication consisted of intramuscular clonidine 2 μg/kg administered both the evening before surgery and 30 min preoperatively to provide anxiolysis and preemptive analgesia. Clonidine, a selective centrally acting partial *α*2‐agonist, has been reported to be a useful preanesthesia medication because of its sedative and analgesic properties [4].

### 2.2. Intraoperative Management

Given the anatomical and functional airway challenges, awake fiberoptic intubation was selected as the primary approach. HFNO was initiated early to ensure adequate preoxygenation using the Optiflow system (Fisher & Paykel Healthcare Limited, Auckland, New Zealand), delivering oxygen at 50 L/min with FiO_2_ 1.0, heated and humidified to 37°C. The patient was positioned semisitting to reduce diaphragmatic splinting and preserve functional residual capacity, while HFNO was initiated early using the Optiflow system to ensure adequate preoxygenation. Minimal sedation was achieved with remifentanil target‐controlled infusion (TCI) at 1‐2 μg/mL using the Minto model, allowing successful fiberoptic intubation without complications.

Following confirmation of proper tube placement, general anesthesia was induced using propofol TCI (Schneider model: 4 μg/mL) and remifentanil TCI (Minto model: 4 μg/mL). Anesthesia was maintained with propofol (2.3–3.5 μg/mL) and remifentanil (4–6 μg/mL) without neuromuscular blockade. Regional anesthesia was provided through bilateral alveolar nerve blocks using mepivacaine 2% with epinephrine 1:100,000. The surgical approach to extract multiple dental elements utilized atraumatic extraction techniques with manual instruments to minimize bleeding risk from angiomatous vessels, avoiding the use of rotating machinery and employing only extractors, forceps, and levers to reduce the risk of ongoing bleeding. After the surgical procedure, resorbable membranes and sutures were used to close the surgical defect. Continuous monitoring included vital signs assessment, Bispectral Index (BIS) monitoring targeting values between 40 and 60, and careful blood loss evaluation throughout the procedure [5]. Propofol and remifentanil were discontinued 10 and 5 min before the extubation assessment, respectively. The patient demonstrated adequate consciousness and command‐following before successful extubation in a semisitting position.

### 2.3. Postoperative Course and Outcomes

PACU management initially involved attempted CPAP therapy, which proved intolerable due to facial discomfort, resulting in significant desaturation to SpO_2_ 80%. HFNO therapy was immediately initiated as a rescue intervention, delivered via nasal cannula at flow rates of 50–70 L/min with FiO_2_ ranging from 60% to 80%. Ventilatory adequacy was actively assessed through repeated clinical evaluations and arterial blood gas analysis during postoperative monitoring to exclude progressive hypercapnia. Postoperative pain was managed using a multimodal, opioid‐sparing analgesic regimen to minimize respiratory depression and improve tolerance to noninvasive respiratory support.

The HFNO intervention achieved rapid improvement in oxygenation within 3 min, with SpO_2_ returning to > 94%. Following 24 h of continuous respiratory support and comprehensive monitoring, the patient met discharge criteria with a postanesthetic discharge score (PADS) ≥ 9. Discharge management emphasized multimodal analgesia without long‐acting opioids, utilizing acetaminophen and NSAIDs. Comprehensive patient education addressed CPAP compliance importance and recognition of delayed complications. The anesthesiology team conducted a thorough reassessment before home discharge, ensuring appropriate transition of care with instructions regarding vigilance for delayed exacerbation of hypersomnolence.

### 2.4. Pre‐ and Postoperative Psychological Support

A personalized psychological support program was implemented to prepare the patient for surgery, foster therapeutic relationships with the care team, and enhance treatment adherence. Continued psychological support for 3 months addressed physical and psychological adaptations, body image concerns, and identity adjustments. Family involvement and relationship network support were integral components of the comprehensive care approach.

## 3. Discussion

This case report underscores the complex perioperative management required for patients presenting with the rare combination of SWS and OHS. This clinical scenario entails multiple interrelated challenges. Specifically, angiomatous airway lesions increase the risk of both hemorrhage and difficult intubation. At the same time, obesity‐associated respiratory compromise further elevates the risk of perioperative complications. Moreover, in patients with OHS, hypoventilation and hypercapnia may occur independently of hypoxemia, and reliance on peripheral oxygen saturation alone is insufficient to assess ventilatory adequacy in the perioperative period. Furthermore, alternative interfaces, including total face masks and nasal pillows, were considered; however, their use was limited by facial angiomatosis and patient discomfort related to SWS. Therefore, this case illustrates that meticulous preoperative planning and evidence‐based strategies can lead to successful outcomes, even in high‐risk contexts.

Patients with SWS often require multiple interventions throughout life, including dental surgeries, trabeculectomy, ocular evaluations, and seizure‐related procedures [3], all of which frequently necessitate anesthesia. In these patients, airway management is paramount, as vascular malformations may render standard intubation unsafe. Awake fiberoptic intubation remains the gold standard for patients with anticipated difficult airways due to angiomatous involvement [6, 7]. This technique minimizes trauma to fragile vascular structures while securing the airway before the induction of general anesthesia.

Respiratory management presents an added layer of complexity. Among the potential strategies, while CPAP primarily acts by maintaining upper airway patency, noninvasive ventilation (NIV) provides additional inspiratory pressure support and is specifically indicated when hypoventilation and hypercapnia are present, as frequently occurs in OHS. Therefore, CPAP and NIV represent distinct therapeutic modalities with different physiological targets and should not be used interchangeably. Nonetheless, while CPAP is considered the gold standard treatment for OHS [8], it is often poorly tolerated. Indeed, nonadherence rates for CPAP range between 29% and 83%, posing a substantial clinical challenge [9–12]. In such cases, HFNO can be implemented as a temporary rescue strategy. HFNO supports adequate oxygenation, reduces dead space, and provides low‐level positive airway pressure. In our case, the patient, unable to tolerate CPAP, responded favorably to HFNO, which prevented respiratory decompensation and the need for reintubation. The rapid improvement in oxygenation within three minutes of HFNO initiation supports its efficacy as rescue therapy in CPAP‐intolerant patients [12].

Importantly, this case emphasizes the role of a multidisciplinary approach, involving anesthesiology, sleep medicine, psychology, and surgical teams. Effective coordination among these specialties is essential when managing patients with multisystem comorbidities such as SWS and OHS. Furthermore, integrating psychological support throughout the perioperative journey can enhance patient engagement and adherence, particularly in cases involving chronic disease management and prior negative healthcare experiences.

### 3.1. Clinical Implications

This case provides valuable insights into the safe anesthetic and perioperative management of patients with complex comorbidities. It highlights the importance of individualized planning and adapting standard protocols to patient‐specific factors. The use of HFNO in patients unable to tolerate CPAP may serve as a temporary rescue or bridging strategy when positive airway pressure is not tolerated, under strict ventilatory monitoring. Nevertheless, HFNO should not be considered an alternative to CPAP or NIV, which differ in their mechanisms and indications, in patients with OHS, as it does not provide ventilatory support and may mask hypoventilation. Although this strategy effectively improves oxygenation by increasing FiO_2_, it does not correct hypoventilation and may delay the recognition of hypercapnia. Thus, when HFNO is used in patients with OHS, monitoring of ventilation through arterial blood gases, end‐tidal CO_2_, or transcutaneous CO_2_ is essential to prevent occult hypercapnia (Table 1).

**Table 1:** Perioperative challenges and management strategies.

Perioperative challenge	Mechanism or risk	Management strategy	Involved specialist (s)
Oral and airway angiomatosis	High risk of bleeding and difficult airway	Awake fiberoptic intubation, atraumatic anesthesia, conservative surgical approach	Anesthesiologist, surgeon
Obesity and OHS	Hypoxemia, hypercapnia, reduced ventilatory response	HFNO, blood gas monitoring, semisitting positioning	Pulmonologist, anesthesiologist
Poor CPAP tolerance	Discontinuation of noninvasive ventilation post‐op	HFNO as rescue therapy, close SpO_2_ monitoring	Anesthesiologist, respiratory therapist
History of tracheostomy due to hemorrhage	High risk in case of emergency reintubation or bleeding	Experienced difficult airway team, emergency backup plan	Anesthesiologist, ENT specialist
Neurological risk in SWS	Seizures or stroke‐like episodes due to instability	Avoid hypoxia and hypercapnia, ensure hemodynamic and analgesic stability	Neurologist, anesthesiologist
Poor compliance and perioperative anxiety	Reduced adherence to instructions, anxiety‐driven disconnection from support	Pre‐ and postoperative psychological support, anxiolysis with clonidine	Psychologist, anesthesiologist
Sedation and analgesia	Risk of respiratory depression	Avoid long‐acting opioids, multimodal analgesia, and brain monitoring	Anesthesiologist, pain specialist

Abbreviations: CPAP, continuous positive airway pressure; HFNO, high‐flow nasal oxygen; OHS, obesity hypoventilation syndrome; SWS, Sturge–Weber syndrome.

### 3.2. Limitations

While informative, this report carries inherent limitations. The rarity of the SWS–OHS combination limits its generalizability. Management strategies may vary depending on institutional resources, the severity of angiomatous lesions, and the extent of respiratory dysfunction. Moreover, longitudinal outcomes and the incidence of delayed postoperative complications were not captured and require further study. Finally, although HFNO proved effective in this instance, larger studies and controlled trials are necessary to establish robust evidence supporting its use in the perioperative setting for CPAP‐intolerant OHS patients.

## 4. Conclusions

The treatment and anesthetic management of patients with SWS and OHS—particularly those compliant with NIV—require a comprehensive, individualized, and interdisciplinary approach to effectively address complex clinical challenges and improve both perioperative safety and long‐term outcomes. Advanced airway skills are mandatory to minimize the risk of injury, especially in the presence of fragile angiomatous airway structures. Based on our experience, we recommend awake fiberoptic intubation as the preferred and most reliable strategy for securing the airway in these high‐risk patients, particularly those with OHS, where standard techniques may fail. Although HFNO may not replace CPAP therapy as the first‐line modality, it can serve as a temporary rescue or bridging strategy in the immediate postoperative period when CPAP is poorly tolerated or infeasible. Its prompt application may help prevent respiratory deterioration and reduce the need for reintubation. Nevertheless, in the presence of persistent or worsening hypercapnia, escalation to NIV providing inspiratory pressure support represents the appropriate alternative to CPAP.

**Nomenclature**
BMI: Body Mass Index
BIS: Bispectral Index
CPAP: Continuous positive airway pressure
ESS: Epworth Sleepiness Scale
FiO2: Fraction of inspired oxygen
HFNO: High‐flow nasal oxygen
NSAIDs: Nonsteroidal anti‐inflammatory drugs
OHS: Obesity hypoventilation syndrome
OSAS: Obstructive sleep apnea syndrome
PaCO2: Partial pressure of carbon dioxide in arterial blood
PADS: Postanesthetic Discharge Score
PaO2: Partial pressure of oxygen in arterial blood
SpO2: Peripheral oxygen saturation
SWS: Sturge–Weber syndrome
TCI: Target‐controlled infusion
V1: Ophthalmic division of trigeminal nerve
V2: Maxillary division of trigeminal nerve

## Author Contributions

Giuseppe Mincolelli conceived the study, designed the protocol, contributed to data acquisition, and drafted the manuscript. Antonio Izzi and Matteo Luigi Giuseppe Leoni participated in data collection and critically reviewed the manuscript. Vincenzo Marchello and Maria Grazia Di Carlo assisted in data interpretation and editing of the final version. Marco Cascella and Andreaserena Recchia contributed to the manuscript revision and literature review. Ruggero Massimo Corso supervised the project, provided clinical oversight, and approved the final manuscript.

## Funding

This research received no external funding. Open access publishing facilitated by Universita degli Studi di Salerno, as part of the Wiley ‐ CRUI‐CARE agreement.

## Disclosure

All authors read and approved the final version of the manuscript.

## Ethics Statement

The authors have nothing to report.

## Consent

The patient provided written consent for publication to the authors.

## Conflicts of Interest

The authors declare no conflicts of interest.

## Data Availability

The data that support the findings of this study are available from the corresponding author upon reasonable request.
